# Case report: Clinical features of COVID-19 vaccine-induced exacerbation of psoriasis–A case series and mini review

**DOI:** 10.3389/fmed.2022.995150

**Published:** 2022-09-26

**Authors:** Sascha Ständer, Henner Zirpel, Florin Bujoreanu, Alin Laurentiu Tatu, Ralf J. Ludwig, Diamant Thaçi

**Affiliations:** ^1^Research Institute and Comprehensive Center for Inflammation Medicine, University of Lübeck, Lübeck, Germany; ^2^Department of Dermatology, University of Lübeck, Lübeck, Germany; ^3^Department of Dermatology, “Sf. Parascheva” Infectious Diseases Clinical Hospital, Galati, Romania; ^4^Department of Clinical Medical, Faculty of Medicine and Pharmacy, “Dunărea de Jos” University of Galaţi, Galaţi, Romania; ^5^Multidisciplinary Integrated Center of Dermatological Interface Research Center (MICDIR), “Dunărea de Jos” University of Galaţi, Galaţi, Romania; ^6^Lübeck Institute of Experimental Dermatology (LIED), University of Lübeck, Lübeck, Germany

**Keywords:** psoriasis, vaccination, COVID-19, new-onset, exacerbation (symptom flare up)

## Abstract

Recently, COVID-19 vaccination-induced exacerbation or new-onset of psoriasis have been reported. Underlying immune pathogenesis is unclear and different mechanisms are assumed. Further, clinical- and vaccine-related features and characteristics are partly inconsistent and remain to be elucidated. To add to the understanding of COVID-19 vaccination-triggered psoriasis, we report five cases with exacerbation or new-onset of psoriasis. In our cohort, one patient experienced the new onset of psoriasis, while four had an exacerbation following COVID-19 vaccination. In most patients, exacerbation or new onset occurred after the 2nd or 3rd vaccination. The mean latency from the day of vaccination was 7.2 (1.8) days (SD). The clinical impact with a mean PASI increase following COVID-19 vaccination of 7.2 (5.6) was considered relevant. In most cases, psoriatic lesions almost cleared after applying topical steroids in addition to current treatment, while one patient with psoriatic arthritis required systemic treatment. New onset and exacerbation of psoriasis have also been noted following COVID-19 infections. Hence, the underlying inflammatory response is most likely the culprit agent triggering psoriasis. This underscores that the benefits of COVID-19 vaccination far outweigh the risks, as also in patients with psoriasis.

## Introduction

Psoriasis is a chronic, immune-mediated inflammatory skin disease that may be induced by both local factors, also known as the Koebner phenomenon, and systemic triggers, i.e., streptococcal infections (1). Vaccine-related onset or exacerbations of psoriasis in patients with precipitated influenza or tetanus-diphtheria vaccination have been described (2, 3). To control the current COVID-19 pandemic, respective vaccinations are being carried out worldwide. Recently, several cases of psoriasis exacerbation (as measured by an increased PASI and/or BSA score), preceded by COVID-19 vaccination emerged (4–6). However, current knowledge on features of COVID-19 vaccine-induced psoriasis worsening is inconsistent and remains to be firmly established. Thus, we aim to add further insight into COVID-19 vaccine-related characteristics of psoriasis patients that presented with a COVID-19 vaccine-induced exacerbation. Specifically, we aim to elucidate latency from vaccination to manifestation of clinical worsening, a dose of vaccination, and clinical features.

## Case description

The current case series comprises five patients that presented at our department with new onset or exacerbation of psoriasis, following COVID-19 vaccination, of whom 2 (40%) were women and 3 (60%) were men. Before the consultation, all patients were free of clinical COVID-19-specific symptoms and were tested negative for COVID-19 infection by PCR. The mean age (SD) at presentation was 51.8 (10.4) years. Four patients (80%) had a known history of psoriasis and 3 (60%) had concomitant psoriatic arthritis (PsA). Of note, three (60%) patients received systemic biologic treatment before vaccination. One patient had new onset of psoriasis and PsA after the vaccination. The most frequently used vaccine was BNT162b2 mRNA (Pfizer-BioNTech) in 4 patients (80%), followed by mRNA-1273 (Moderna) vaccine in 1 patient (20%). Four patients (80%) reported exacerbation or new onset of psoriasis after 2nd or 3rd dose, while one patient (20%) had exacerbation after receiving the 1st dose. Mean latency from the day of vaccination until exacerbation or new onset accounted for 7.2 (1.8) days. Clinically, three (60%) patients presented with mostly plaque-type psoriasis, while two (40%) patients displayed small papules and plaques clinical features. Mean PASI increase was documented after a vaccine-induced psoriasis worsening was documented at 7.2 (5.6), an increase of the total body surface (BSA) was 9.6 (8.9), and a worsening of the Dermatology Life Quality Index (DLQI) score was 12.8 (7.6). Interestingly, most patients reported a mild-to-moderate itch of 2 (2.1) /10 numeric rating scale (NRS). Patients' main characteristics are summarized in Table 1.

**Table 1:** Clinical and vaccine-related characteristics of 5 COVID-19 vaccine-related psoriasis cases.

**Patient**	**Sex**	**Age (years)**	**New onset**	**Lantency (days)**	**Vaccine**	**Dose**	**Type**	**Localization**	**PASI (before)**	**PASI increase**	**BSA % (before)**	**BSA increase**	**DLQI (before)**	**DLQI increase**	**Pruritus**	**PsA**	**Treatment**
1	m	35	no	7	Comirnaty	2nd	Guttate	Extremities	16,9 (0)	16,9	25 (0)	25	25 (n.a.)	25	2	yes	no
2	f	50	yes	7	Comirnaty	2nd	Plaque	Head	4,5 (0)	4,5	7 (0)	7	15 (n.a.)	15	3	yes	no
3	m	62	no	10	Moderna	2nd	Guttate	Trunk	5,0 (1, 5)	3,5	11 (4)	9	12 (5)	7	0	yes	Ustekinumab
4	f	58	no	5	Comirnaty	2nd	Plaque	Palmoplantar	5,4 (1, 2)	4,2	3 (1)	2	26 (19)	7	0	no	Ixekizumab
5	m	54	no	7	Comirnaty	3rd	Plaque	Extremities	11,9 (4, 7)	7	10 (5)	5	12 (2)	10	5	no	Ixekizumab

## Diagnostic assessment

Patient 1 is a 35-year-old man with a history of psoriasis and psoriatic arthritis with no symptoms during the last 4 years. At presentation, he was without any psoriasis treatment. Seven days after the 2nd dose of BNT162b2 mRNA (Pfizer-BioNTech) vaccine, psoriasis recurred with a predominant eruptive exanthematic clinical presentation. Additionally, pains in the proximal interphalangeal (PIP) joints of both hands were reported. No swollen or tender joints were detected. After topical treatment with betamethasone/calcipotriol ointment, almost all cutaneous lesions cleared. However, joint affection remained persistent under treatment with 60 mg of etoricoxib once a day. Currently, diagnostic workup of joint pain is ongoing.

Patient 2, a 50-year-old woman with no prior reported psoriatic skin lesions, reported onset of psoriasis 7 days after the 2nd dose of BNT162b2 mRNA (Pfizer-BioNTech). Main plaque-type involvement of the scalp with few solitary plaques on the trunk and extremities were the leading clinical features. Pain and swelling were present in all the PIP joints, and a PsA was diagnosed according to the Classification Criteria for Psoriatic Arthritis (CASPAR). Similar to patient 1, skin lesions cleared after topical treatment with betamethasone/calcipotriol ointment. Due to persisting joint pain and swelling, 15 mg/week of methotrexate was initiated.

**Figure 1:**
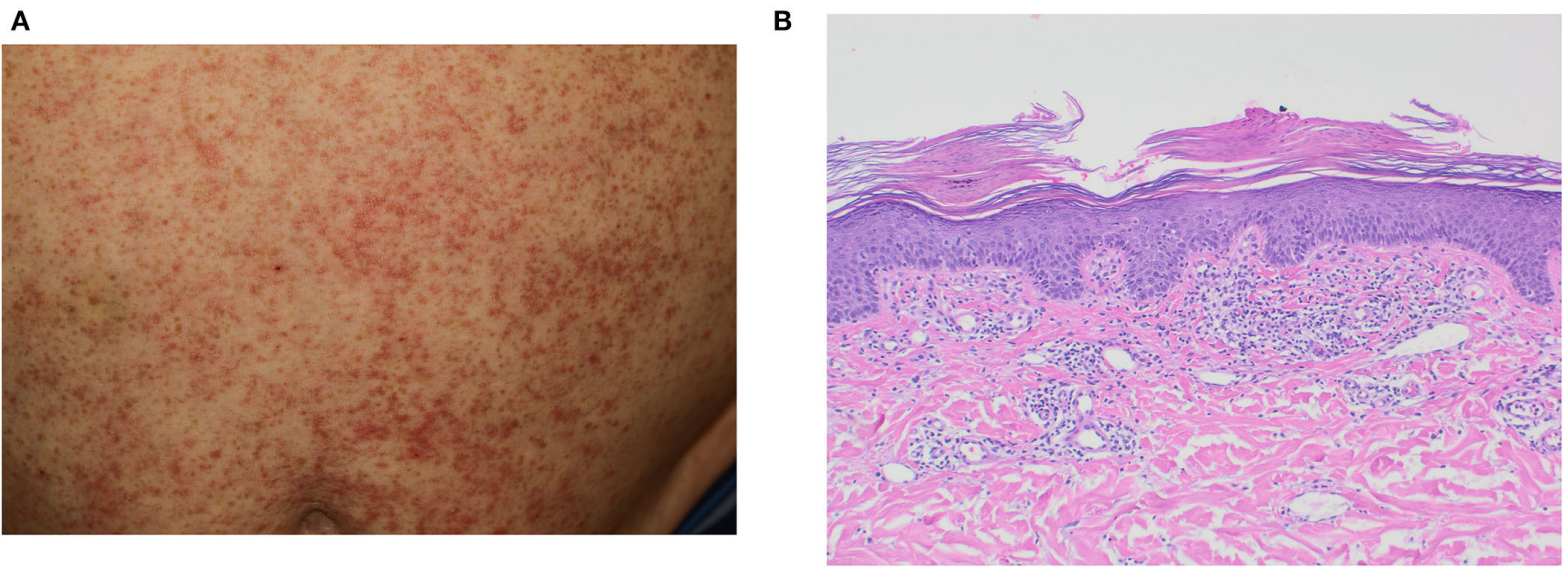
Clinical and histopathological presentation of patient 3. **(A)** guttate-like sharply confined erythematosquamous papules, **(B)** parakeratosis and acanthosis with a patch-like lymphohistiocytic inflammatory infiltrate in histopathology.

Patient 3, a 62-year-old man, displayed worsening psoriasis currently treated with the p40 (IL-12/23) antibody ustekinumab. Exacerbation occurred with a latency of 10 days after 2nd injection of mRNA-1273 (Moderna) and led to eruptive exanthematic (guttata-like) affection of mainly the trunk (Figure 1A). Dermatohistopathological examination revealed a parakeratosis and acanthosis, with a patch-like lymphohistiocytic inflammatory infiltrate that was suggestive of psoriasis (Figure 1B). Interestingly, no itch was reported, and previously diagnosed psoriatic arthritis remained clinically silent. Due to persistent skin lesions under ustekinumab, treatment was switched to guselkumab. Within several weeks, significant improvement in skin lesions was noted.

Patient 4, a 58-year-old woman, received the 2nd BNT162b2 mRNA (Pfizer-BioNTech) and developed an exacerbation of a mainly palmoplantar localized psoriasis after 5 days. Regarding subjective complaints, the patient reported no itch. During this time, the patient was under treatment with the IL-17A inhibitor ixekizumab. The patient's skin lesions quickly responded to an intensified topical application of topical corticostoid treatment; initially, clobetasol was followed by betamethasone/calcipotriol once per day.

Patient 5, a 54-year-old man, presented with worsening of psoriasis under s.c. treatment with IL-17A inhibitor ixekizumab after the 3rd injection of BNT162b2 mRNA (Pfizer-BioNTech) with a latency of 7 days. In addition, he reported a slight worsening of his skin condition after the 2nd vaccination. The patient displayed predominant affection for extremities and umbilicus with a moderate itch of 5/10 on the NRS and a plaque-type clinical manifestation. Psoriatic skin lesion improved after adding topical treatment with betamethasone/calcipotriol ointment once a day.

## Discussion

The current case series reports four cases with an exacerbation, and one case with new onset of psoriasis and psoriatic arthritis, following vaccination with two different COVID-19 vaccines. To the best of our knowledge, 75 cases of individuals with exacerbation and 15 patients with new onset psoriasis, after vaccination had been reported, so far (Supplementary Table 1). Furthermore, other reports of development/exacerbation of inflammatory/psoriatic arthritis after COVID-19 vaccination had been reported in the literature (8, 9). The mean latency from vaccine injection to the manifestation of a clinical exacerbation and new onset accounted for approximately 14 (10.9) days in 68 reported cases, which is approximately in line with our findings of latency of 7 days since exacerbation, or new onset are reported in 81% of cases within the first 3 weeks (Supplementary Table 1). Similarly, unspecific local triggers can cause an isomorphic cutaneous reaction in a similar time, also known as the Koebner phenomenon. Here, we found that most flare-ups were reported after 2nd vaccination. This is in line with the current literature, showing that the majority of psoriasis inductions occur after 2nd vaccination (61%). While the most encountered vaccine in this case series was BNT162b2 mRNA (Pfizer-BioNTech), reasoned by its high predominance of usage in Germany, reported vaccines in literature are mRNA−1273 (Moderna) in 18 cases, AZD1222 (AstraZeneca) in 24 cases, BNT162b2 mRNA (Pfizer-BioNTech) in 40 cases, Sinovac (CoronaVac) in four cases, and Covaxin in one case (Supplementary Table 1). Further, in a registry-based study by McMahon et.al., 414 cutaneous reactions were analyzed. It was reported that in 63% of patients receiving the first and second dose of vaccination, a cutaneous reaction after the second dose was reported (7). Among these, delayed large local reactions, local injection site reactions, urticarial eruptions, and morbilliform eruptions were the most common. Whether psoriasis exacerbation is mainly caused after 2nd dose, pointing toward increased immune response, needs further investigation, especially considering upcoming booster vaccinations.

Most patients in this case series showed an improved skin condition upon intensified topical anti-inflammatory treatment, while in one case, systemic treatment needed to be changed. Thus, cutaneous worsening of psoriasis is mostly managed by local anti-inflammatory treatment, while induction of PsA presumably requires an interdisciplinary dermatological/rheumatological approach and systemic treatment.

The pathogenesis of vaccination-mediated psoriasis has not been resolved yet. However, psoriasis exacerbation after vaccination had been reported for influenza and tetanus-diphtheria vaccination (2, 3, 10). Increased levels of IL-6 were measured after administration of the latter one, which is known to promote TH17 skewing. Vaccination with mRNA vaccine ChAdOx1 nCoV-19 (AZD1222) resulted in increased levels of IL-2, IL-12, TNF-α, and INF-γ in healthy individuals (11). Type 1 interferon (INF-1) expression occurs in peripheral dendritic cells (pDCs) upon activation *via* TLR-7, which is known to bind nucleic acids. It was reported that mRNA vaccines interact with endosomal TLRs, such as TLR7 (12). Compartmentalization of SARS-CoV-2 particles by pDCs results in type 1 INF cytokine expression (13). Thus, it can be speculated that binding of mRNA *via* TLR7 and followed increase of TH1 cytokines could result in worsening or new onset of psoriasis in genetically prone individuals.

Beside binding of nucleic acid *via* TLR7, the vaccine vector can be immunostimulatory in a few individuals, as extensively reviewed by Shirley et. al., resulting in increased IFN-1 response (14). This possibility is supported by findings that increased levels of TNF-α and IFN-γ were found after AZD1222 vaccination. However, from all 90 reported cases, so far, only 24 individuals were vaccinated using a vector system vaccine (AZD1222). In contrast, vaccines, such as mRNA-1273 and mRNABNT132b2, use lipid nanoparticles. Therefore, INF-1 response resulting from vector immunostimulation might account for only a few cases. However, all the above may also be triggered by the corresponding infection. More specifically, 47 cases of COVID-19 infection exacerbation / new onset of psoriasis have been reported (Supplementary Table 2). Given that by July 2022, ~544 million people worldwide had been infected with the SARS-CoV-2 virus and around 4.7 billion people are currently vaccinated with at least two doses of COVID-19 vaccine, onset or exacerbation of psoriasis in vaccinated individuals (1.88 ^*^ 10^−6^%) is reduced compared to individuals with infection (8.64 ^*^ 10^−6^%). Hence, the benefit of vaccination outweighs the risk of triggering psoriasis.

Overall, this case series adds to the growing number of psoriasis' new onset or exacerbation cases. The above-discussed possible mechanisms of onset or exacerbation might reason individual cases and provide new clues to psoriasis pathogenesis in the future.

## Data availability statement

The original contributions presented in the study are included in the article/Supplementary material, further inquiries can be directed to the corresponding author.

## Ethics statement

Ethical review and approval was not required for the study on human participants in accordance with the local legislation and institutional requirements. The patients/participants provided their written informed consent to participate in this study. Written informed consent was obtained from the individual(s) for the publication of any potentially identifiable images or data included in this article.

## Author contributions

Conceptualization and investigation: SS, HZ, RL, and DT. Methodology: SS, HZ, and RL. Validation: FB, AT, RL, and DT. Formal analysis, resources, and data curation: SS and HZ. Project administration and funding acquisition: DT. writing—original draft preparation, writing—review and editing, and visualization: all authors. All authors have read and agreed to the published version of the manuscript.

## Funding

This work has been financially supported by the Cluster of Excellence Precision Medicine in Chronic Inflammation (EXC 2167) from the Deutsche Forschungsgemeinschaft and the Schleswig-Holstein Excellence-Chair Program from the State of Schleswig Holstein. The APC was paid by “Dunarea de Jos” University of Galati which also academically supported this paper through the Multidisciplinary Integrated Center of Dermatological Interface Research (MIC-DIR).

## Conflict of interest

Over, the past 3 years, RL has received honoraria and/or research grants from the following companies: Admirx, Almirall, Amryth, ArgenX, Biotest, Biogen, Euroimmun, Incyte, Immungenetics, Lilly, Novartis, UCB Pharma, Topadur, True North Therapeutics, and Tx Cell. DT has received honoraria or fees for serving on advisory boards, as a speaker, as a consultant from AbbVie, Amgen, Almirall, Beiersdorf, Bristol-Meiers-Squibb, Boehringer Ingelheim, Galapagos, Leo PharmaMerck Sharp & Dohme, Morphosys, Lilly, Novartis, Janssen-Cilag, Pfizer, Regeneron, Sanofi, Hexal, Sun Pharmaceuticals, and UCB and grants from Leo and Novartis. The remaining authors declare that the research was conducted in the absence of any commercial or financial relationships that could be construed as a potential conflict of interest.

## Publisher's note

All claims expressed in this article are solely those of the authors and do not necessarily represent those of their affiliated organizations, or those of the publisher, the editors and the reviewers. Any product that may be evaluated in this article, or claim that may be made by its manufacturer, is not guaranteed or endorsed by the publisher.
