# Supplementary material for: Case report: Clinical features of COVID-19 vaccine-induced exacerbation of psoriasis–A case series and mini review
Source: Front Med (Lausanne). 2022 Sep 26;9:995150. doi: 10.3389/fmed.2022.995150 (PMC9549869; doi:10.3389/fmed.2022.995150)
Supplement: Supplementary file 1 [file Data_Sheet_1.docx]

Supplementary Material

# Supplementary Figures and Tables

| 1st Author | # of cases (total) | new-onset | exacerbation | Moderna (mRNA-1273) | AZ (AZD1222) | Pfizer (mRNABNT162b2) | Sinovac (CoronaVac) | Covaxin (BBV152) | 1st vaccination | 2nd vaccination | 3rd vaccination | Latency [days] |
| --- | --- | --- | --- | --- | --- | --- | --- | --- | --- | --- | --- | --- |
| Bostan, *et.al*.(1) | 2 | 0 | 2 |  |  | 2 |  |  | 1 | 1 |  | 30, 60 |
| Chhabra, *et.al.*(2) | 1 | 1 | 0 |  | 1 |  |  |  | 1 |  |  | 14 |
| Durmaz, *et.al.*(3) | 3 | 0 | 3 |  |  | 3 |  |  | 1 | 1 | 1 | 3, 7, 42 |
| Durmus, *et.al.*(4) | 1 | 0 | 1 |  |  | 1 |  |  | 1 |  |  | 28 |
| Elamin, *et.al.*(5) | 1 | 1 | 0 |  | 1 |  |  |  | 1 |  |  | 21 |
| Fang, *et.al.*(6) | 1 | 0 | 1 |  | 1 |  |  |  | 1 |  |  | 14 |
| Frioui, *et.al.*(7) | 1 | 1 | 0 |  |  | 1 |  |  | 1 |  |  | 4 |
| Huang, *et.al.*(8) | 17 | 0 | 17 | 7 | 8 |  |  |  |  |  |  |  |
| Infimate, *et.al.*(9) | 1 | 0 | 1 |  |  |  |  | 1 | 1 |  |  | 4 |
| Kabbani, *et.al.*(10) | 1 | 0 | 1 |  |  | 1 |  |  |  | 1 |  | 7 |
| Krajewski, *et.al.*(11) | 1 | 0 | 1 |  |  | 1 |  |  |  | 1 |  | 5 |
| Lehmann, *et.al.*(12) | 1 | 1 | 0 |  |  | 1 |  |  | 1 |  |  | 10 |
| Lopez, *et.al.*(13) | 1 | 0 | 1 |  |  | 1 |  |  |  | 1 |  | 4 |
| Megna, *et.al.*(14) | 11 | 0 | 11 | 1 | 3 | 7 |  |  | 2 | 9 |  | 5, 5, 6, 7, 8, 8, 9, 10,10, 12, 14 |
| Mieczkowska, *et.al.*(15) | 1 | 0 | 1 |  |  | 1 |  |  | 1 |  |  | 7 |
| Nagrani, *et.al.*(16) | 2 | 1 | 1 |  | 2 |  |  |  |  | 2 |  | 2, 10 |
| Nia, *et.al.*(17) | 1 | 0 | 1 |  |  | 1 |  |  |  |  |  | 1 |
| Onsun, *et.al.*(18) | 1 | 0 | 1 |  |  |  | 1 |  | 1 |  |  | 4 |
| Pavia, *et.al.*(19) | 1 | 0 | 1 |  |  | 1 |  |  |  | 1 |  | 10 |
| Perna, *et.al.*(20) | 1 | 0 | 1 |  |  | 1 |  |  | 1 |  |  | 5 |
| Pesque, *et.al.*(21) | 2 | 1 | 1 | 2 |  |  |  |  | 1 | 1 |  | 6, 10 |
| Phuan, *et.al.*(22) | 1 | 0 | 1 |  |  | 1 |  |  |  |  | 1 | 7 |
| Piccolo, *et.al.*(23) | 2 | 0 | 2 |  |  |  |  |  |  |  |  | 30 |
| Quattrini, *et.al.*(24) | 1 | 0 | 1 |  |  | 1 |  |  |  | 1 |  | 2 |
| Ricardo, *et.al.*(25) | 1 | 1 | 0 |  |  | 1 |  |  |  |  |  |  |
| Romagnuolo, *et.al.*(26) | 1 | 1 | 0 |  |  | 1 |  |  | 1 |  |  |  |
| Shakoei, *et.al.*(27) | 3 | 2 | 1 |  |  |  | 3 |  | 1 | 2 |  | 7 |
| Song, *et.al.*(28) | 1 | 1 | 0 |  |  | 1 |  |  | 1 |  |  | 2 |
| Sotiriou, *et.al.*(29) | 14 | 0 | 14 | 1 | 7 | 6 |  |  | 2 | 12 |  | 3, 5, 6, 6, 7, 7, 7, 8, 9, 10, 10, 13, 22, 32 |
| Tachibana, *et.al.*(30) | 2 | 0 | 2 |  |  | 2 |  |  | 1 | 1 |  | 7, 8 |
| Tran, *et.al.*(31) | 3 | 3 | 0 | 1 | 1 | 1 |  |  | 2 |  | 1 | 7, 30, 30 |
| Tran, *et.al.*(32) | 2 | 0 | 2 |  |  | 2 |  |  |  | 2 |  | 7, 7 |
| Wei, *et.al.*(33) | 7 | 1 | 6 | 6 |  | 1 |  |  | 1 | 6 |  | 6, 7, 21, 24, 60, 62, 90 |
| Yatsuzuka, *et.al.*(34) | 1 | 0 | 1 |  |  | 1 |  |  |  | 1 |  | 12 |
|  | **91** | **15** | **76** | **18** | **24** | **40** | **4** | **1** | **24** | **43** | **3** | **Ø 14,31** |

supplementary Table 1. Current case reports of exacerbation or new-onset of psoriasis after Covid-19 vaccination.

| 1st Author | # of cases (total) | new-onset | exacerbation |
| --- | --- | --- | --- |
| Abadie, *et.al.*(35) | 1 | 0 | 1 |
| Batubara, *et.al.*(36) | 1 | 0 | 1 |
| Carugno, *et.al.*(37) | 1 | 0 | 1 |
| Dadras, *et.al.*(38) | 1 | 0 | 1 |
| Demiri, *et.al.*(39) | 1 | 0 | 1 |
| Essien, *et.al.*(40) | 1 | 1 | 0 |
| Gananandan, *et.al.*(41) | 1 | 0 | 1 |
| Ghalamkarpour, *et.al.*(42) | 1 | 0 | 1 |
| Janodia, *et.al.*(43) | 1 | 1 | 0 |
| Kutlu, *et.al.*(44) | 1 | 0 | 1 |
| Mroz, *et.al.*(45) | 14 | 0 | 14 |
| Nasiri, *et.al.*(46) | 1 | 0 | 1 |
| Ohmura, *et.al.*(47) | 1 | 0 | 1 |
| Ozaras, *et.al.*(48) | 1 | 0 | 1 |
| Pala, *et.al.*(49) | 1 | 0 | 1 |
| Polat, *et.al.*(50) | 15 | 0 | 15 |
| Rouai, *et.al.*(51) | 1 | 1 | 0 |
| Samotij, *et.al.*(52) | 1 | 1 | 0 |
| Shakoei, *et.al.*(53) | 1 | 0 | 1 |
| Sigha, *et.al.*(54) | 1 | 0 | 1 |
|  | **47** | **4** | **43** |

Supplementary table 2. Current case reports of exacerbation of psoriasis after Covid-19 infection.

Literature

1. Bostan E, Elmas L, Yel B, Yalici-Armagan B. Exacerbation of plaque psoriasis after inactivated and BNT162b2 mRNA COVID-19 vaccines: A report of two cases. *Dermatol Ther* (2021) 34: doi: 10.1111/DTH.15110

2. Chhabra N, C AG. A case of de novo annular-plaque type psoriasis following Oxford-AstraZeneca COVID-19 vaccination. *Curr Drug Saf* (2022) 17: doi: 10.2174/1574886317666220613163327

3. Durmaz I, Turkmen D, Altunisik N, Toplu SA. Exacerbations of generalized pustular psoriasis, palmoplantar psoriasis, and psoriasis vulgaris after mRNA COVID-19 vaccine: A report of three cases. *Dermatol Ther* (2022) doi: 10.1111/DTH.15331

4. Durmus O, Akdogan N, Karadag O, Gokoz O. Erythroderma related with the first dose of Pfizer-BioNTech BNT16B2b2 COVID-19 mRNA vaccine in a patient with psoriasis. *Dermatol Ther* (2022) 35:e15363. doi: 10.1111/DTH.15363

5. Elamin S, Hinds F, Tolland J. De novo generalized pustular psoriasis following Oxford-AstraZeneca COVID-19 vaccine. *Clin Exp Dermatol* (2022) 47:153–155. doi: 10.1111/CED.14895

6. Fang WC, Chiu LW, Hu SCS. Psoriasis exacerbation after first dose of AstraZeneca coronavirus disease 2019 vaccine. *J Dermatol* (2021) 48:e566–e567. doi: 10.1111/1346-8138.16137

7. Frioui R, Chamli A, Zaouak A, Hlel I, Khanchel F, Fenniche S, Hammami H. A case of new-onset acute generalized pustular psoriasis following Pfizer-BioNTech COVID-19 vaccine. *Dermatol Ther* (2022) 35:e15444. doi: 10.1111/DTH.15444

8. Huang YW, Tsai TF. Exacerbation of Psoriasis Following COVID-19 Vaccination: Report From a Single Center. *Front Med* (2021) 8: doi: 10.3389/FMED.2021.812010

9. Infimate DL, Yumnam D, Galagali SS, Kabi A, Kaeley N. Psoriasis Flare-Up After COVAXIN BBV152 Whole Virion Inactivated Vaccine. *Cureus* (2022) 14: doi: 10.7759/CUREUS.22311

10. Kabbani M, Poskin M, Benhadou F. Psoriasis exacerbation after COVID-19 vaccination in high-risk group: How to manage it? *Dermatol Ther* (2022) 35:e15368. doi: 10.1111/DTH.15368

11. Krajewski PK, Matusiak, Szepietowski JC. Psoriasis flare‐up associated with second dose of Pfizer‐BioNTech BNT16B2b2 COVID‐19 mRNA vaccine. *J Eur Acad Dermatology Venereol* (2021) 35:e632. doi: 10.1111/JDV.17449

12. Lehmann M, Schorno P, Hunger RE, Heidemeyer K, Feldmeyer L, Yawalkar N. New onset of mainly guttate psoriasis after COVID-19 vaccination: a case report. *J Eur Acad Dermatol Venereol* (2021) 35:e752–e755. doi: 10.1111/JDV.17561

13. Lopez ED, Javed N, Upadhyay S, Shekhar R, Sheikh AB. Acute exacerbation of psoriasis after COVID-19 Pfizer vaccination. *Proc (Bayl Univ Med Cent)* (2022) 35:199. doi: 10.1080/08998280.2021.2003681

14. Megna M, Potestio L, Gallo L, Caiazzo G, Ruggiero A, Fabbrocini G. Reply to “Psoriasis exacerbation after COVID-19 vaccination: report of 14 cases from a single centre” by Sotiriou E et al. *J Eur Acad Dermatol Venereol* (2022) 36:e11–e13. doi: 10.1111/JDV.17665

15. Mieczkowska K, Kaubisch A, McLellan BN. Exacerbation of psoriasis following COVID-19 vaccination in a patient previously treated with PD-1 inhibitor. *Dermatol Ther* (2021) 34: doi: 10.1111/DTH.15055

16. Nagrani P, Jindal R, Goyal D. Onset/flare of psoriasis following the ChAdOx1 nCoV-19 Corona virus vaccine (Oxford-AstraZeneca/Covishield): Report of two cases. *Dermatol Ther* (2021) 34: doi: 10.1111/DTH.15085

17. Nia AM, Silva MM, Spaude J, Gonzalez-Fraga JD. Erythrodermic psoriasis eruption associated with SARS-CoV-2 vaccination. *Dermatol Ther* (2022) 35:e15380. doi: 10.1111/DTH.15380

18. Onsun N, Kaya G, Işık BG, Güneş B. A generalized pustular psoriasis flare after CoronaVac COVID-19 vaccination: Case report. *Heal Promot Perspect* (2021) 11:261–262. doi: 10.34172/HPP.2021.32

19. Pavia G, Gargiulo L, Spinelli F, Avagliano J, Valenti M, Borroni RG, Costanzo A, Narcisi A. Generalized pustular psoriasis flare in a patient affected by plaque psoriasis after BNT162b2 mRNA COVID-19 vaccine, successfully treated with risankizumab. *J Eur Acad Dermatology Venereol* (2022) 36:e502–e505. doi: 10.1111/JDV.18032

20. Perna D, Jones J, Schadt CR. Acute generalized pustular psoriasis exacerbated by the COVID-19 vaccine. *JAAD case reports* (2021) 17:1–3. doi: 10.1016/J.JDCR.2021.08.035

21. Pesqué D, Lopez-Trujillo E, Marcantonio O, Giménez-Arnau AM, Pujol RM. New-onset and exacerbations of psoriasis after mRNA COVID-19 vaccines: two sides of the same coin? *J Eur Acad Dermatol Venereol* (2022) 36:e80–e81. doi: 10.1111/JDV.17690

22. Phuan CZY, Choi EC-E, Oon HH. Temporary exacerbation of pre-existing psoriasis and eczema in the context of COVID-19 messenger RNA booster vaccination: A case report and review of the literature. *JAAD Int* (2022) 6:94–96. doi: 10.1016/J.JDIN.2021.11.004

23. Piccolo V, Russo T, Mazzatenta C, Bassi A, Argenziano G, Cutrone M, Danielsson Darlington MES, Grimalt R. COVID vaccine-induced pustular psoriasis in patients with previous plaque type psoriasis. *J Eur Acad Dermatol Venereol* (2022) doi: 10.1111/JDV.17918

24. Quattrini L, Verardi L, Caldarola G, Peluso G, De Simone C, D’Agostino M. New onset of remitting seronegative symmetrical synovitis with pitting oedema and palmoplantar psoriasis flare-up after Sars-Cov-2 vaccination. *J Eur Acad Dermatol Venereol* (2021) 35:e727–e729. doi: 10.1111/JDV.17502

25. Ricardo JW, Lipner SR. Case of de novo nail psoriasis triggered by the second dose of Pfizer-BioNTech BNT162b2 COVID-19 messenger RNA vaccine. *JAAD case reports* (2021) 17:18–20. doi: 10.1016/J.JDCR.2021.09.009

26. Romagnuolo M, Pontini P, Muratori S, Marzano A V., Moltrasio C. De novo annular pustular psoriasis following mRNA COVID-19 vaccine. *J Eur Acad Dermatology Venereol* (2022) doi: 10.1111/JDV.18114

27. shakoei S, Kalantari Y, Nasimi M, Toutounchi NM, Ansari MS, Razavi Z, Etesami I. Cutaneous manifestations following COVID-19 vaccination: A report of 25 cases. *Dermatol Ther* (2022) doi: 10.1111/DTH.15651

28. Song WJ, Lim Y, Jo SJ. De novo guttate psoriasis following coronavirus disease 2019 vaccination. *J Dermatol* (2022) 49:e30–e31. doi: 10.1111/1346-8138.16203

29. Sotiriou E, Tsentemeidou A, Bakirtzi K, Lallas A, Ioannides D, Vakirlis E. Psoriasis exacerbation after COVID‐19 vaccination: a report of 14 cases from a single centre. *J Eur Acad Dermatology Venereol* (2021) 35:e857–e859. doi: 10.1111/JDV.17582

30. Tachibana K, Kawakami Y, Tokuda M, Sato S, Sugihara S, Miyake T, Sugiura K, Morizane S. Flare-up of generalized pustular psoriasis following Pfizer-BioNTech BNT162b2 mRNA COVID-19 vaccine: Two cases without mutations of IL36RN and CARD14 genes. *J Dermatol* (2022) 00:1–2. doi: 10.1111/1346-8138.16442

31. Nguyen T, Tran A, Thuy |, Phan T, Nguyen N|, Pham N, Nhi |, Pham TU, Thi T, Vu P, et al. New onset of psoriasis following COVID-19 vaccination. *Dermatol Ther* (2022)e15590. doi: 10.1111/DTH.15590

32. Tran TB, Pham NTU, Phan HN, Nguyen HT. Generalized erythrodermic psoriasis triggered by vaccination against severe acute respiratory syndrome Coronavirus 2. *Dermatol Ther* (2022) doi: 10.1111/DTH.15464

33. Wei N, Kresch M, Elbogen E, Lebwohl M. New onset and exacerbation of psoriasis after COVID-19 vaccination. *JAAD case reports* (2022) 19:74–77. doi: 10.1016/J.JDCR.2021.11.016

34. Yatsuzuka K, Murakami M, Kuroo Y, Fukui M, Yoshida S, Muto J, Shiraishi K, Sayama K. Flare-up of generalized pustular psoriasis combined with systemic capillary leak syndrome after coronavirus disease 2019 mRNA vaccination. *J Dermatol* (2022) 49:454–458. doi: 10.1111/1346-8138.16271

35. Abadie MS Al. COVID-19 Infection Cause Moderate-Severe Psoriasis Flare Up. *Eur J Med Heal Sci* (2020) 2: doi: 10.24018/EJMED.2020.2.3.331

36. Batubara IS, Budianti WK. Erythrodermic psoriasis in post-coronavirus disease 2019 patient. *Asia Pac Allergy* (2022) 12: doi: 10.5415/APALLERGY.2022.12.E16

37. Carugno A, Gambini DM, Raponi F, Vezzoli P, Robustelli Test E, Arosio MEG, Callegaro A, Sena P. Coronavirus disease 2019 (COVID‐19) rash in a psoriatic patient treated with Secukinumab: Is there a role for Interleukin 17? *Dermatol Ther* (2020) 33: doi: 10.1111/DTH.14011

38. Shahidi Dadras M, Diab R, Ahadi M, Abdollahimajd F. Generalized pustular psoriasis following COVID‐19. *Dermatol Ther* (2021) 34: doi: 10.1111/DTH.14595

39. Demiri J, Abdo M, Tsianakas A. [Erythrodermic psoriasis after COVID-19]. *Hautarzt* (2021) 73:156–159. doi: 10.1007/S00105-021-04931-0

40. Essien F, Chastant L, McNulty C, Hubbard M, Lynette L, Carroll M. COVID-19-induced psoriatic arthritis: a case report. *Ther Adv Chronic Dis* (2022) 13:204062232210993. doi: 10.1177/20406223221099333

41. Gananandan K, Sacks B, Ewing I. Guttate psoriasis secondary to COVID-19. *BMJ Case Rep* (2020) 13:237367. doi: 10.1136/BCR-2020-237367

42. Ghalamkarpour F, Pourani MR, Abdollahimajd F, Zargari O. A case of severe psoriatic erythroderma with COVID-19. *J Dermatolog Treat* (2020) doi: 10.1080/09546634.2020.1799918

43. Janodia. Guttate Psoriasis Following COVID-19 Infection. *Cutis* (2022) 109: doi: 10.12788/CUTIS.0443

44. Kutlu Ö, Metin A. A case of exacerbation of psoriasis after oseltamivir and hydroxychloroquine in a patient with COVID-19: Will cases of psoriasis increase after COVID-19 pandemic? *Dermatol Ther* (2020) 33: doi: 10.1111/DTH.13383

45. Mroz M, Mućka S, Miodońska M, Ziolkowska D, Hadas E, Bożek A. Influence of SARS-CoV-2 Virus Infection on the Course of Psoriasis during Treatment with Biological Drugs. *Medicina (Kaunas)* (2021) 57: doi: 10.3390/MEDICINA57090881

46. Nasiri S, Araghi F, Tabary M, Gheisari M, Mahboubi-Fooladi Z, Dadkhahfar S. A challenging case of psoriasis flare-up after COVID-19 infection. *J Dermatolog Treat* (2020) 31:448–449. doi: 10.1080/09546634.2020.1764904

47. Ohmura S, Hanai S, Ishihara R, Ohkubo Y, Miyamoto T. A case of psoriatic spondyloarthritis exacerbation triggered by COVID-19 messenger RNA vaccine. *J Eur Acad Dermatology Venereol* (2022) 36:e427–e429. doi: 10.1111/JDV.18013

48. Ozaras R, Berk A, Ucar DH, Duman H, Kaya F, Mutlu H. Covid-19 and exacerbation of psoriasis. *Dermatol Ther* (2020) 33: doi: 10.1111/DTH.13632

49. Pala E, Melikoğlu M, Erkayman MH. Pediatric COVID-19 patient with exacerbated generalized pustular psoriasis. *Rev Soc Bras Med Trop* (2021) 54: doi: 10.1590/0037-8682-0318-2021

50. Kara Polat A, Oguz Topal I, Karadag AS, Aksoy H, Koku Aksu AE, Ozkur E, Ozkok Akbulut T, Topaloglu Demir F, Engin B, Uzuncakmak TK, et al. The impact of COVID-19 in patients with psoriasis: A multicenter study in Istanbul. *Dermatol Ther* (2021) 34: doi: 10.1111/DTH.14691

51. Rouai M, Rabhi F, Mansouri N, Jaber K, Dhaoui R. New-onset guttate psoriasis secondary to COVID-19. *Clin case reports* (2021) 9: doi: 10.1002/CCR3.4542

52. Samotij D, Gawron E, Szczęch J, Ostańska E, Reich A. Acrodermatitis Continua of Hallopeau Evolving into Generalized Pustular Psoriasis Following COVID-19: A Case Report of a Successful Treatment with Infliximab in Combination with Acitretin. *Biologics* (2021) 15:107–113. doi: 10.2147/BTT.S302164

53. Shakoei S, Ghanadan A, Hamzelou S. Pustular psoriasis exacerbated by COVID-19 in a patient with the history of psoriasis. *Dermatol Ther* (2020) 33: doi: 10.1111/DTH.14462

54. Sigha OB, Kouotou EA. Infection à COVID-19 révélée par une poussée de psoriasis chez un Camerounais âgé : à propos d’un cas. *Our Dermatology Online* (2021) 12:16–20. doi: 10.7241/OURD.2021S1.4
